# A Novel Cysteine-Sparing *NOTCH3* Mutation in a Chinese Family with CADASIL

**DOI:** 10.1371/journal.pone.0104533

**Published:** 2014-08-06

**Authors:** Wei Ge, Hanzhe Kuang, Bin Wei, Le Bo, Zhice Xu, Xingshun Xu, Deqin Geng, Miao Sun

**Affiliations:** 1 Institute for Fetology, the First Affiliated Hospital of Soochow University, Suzhou City, Jiangsu, China; 2 Department of Neurology, the Affiliated Hospital of Xuzhou Medical College, Xuzhou City, Jiangsu, China; 3 Department of Neurology, the Second Affiliated Hospital of Soochow University, Suzhou City, Jiangsu, China; Emory University, United States of America

## Abstract

Cerebral autosomal dominant arteriopathy with subcortical infarcts and leukoencephalopathy (CADASIL) is an adult onset cerebral small vessel disorder caused by the mutations of the neurogenic locus notch homolog protein 3 (*NOTCH3*) gene. The extracellular part of NOTCH3 is composed of 34 epidermal growth factor-like (EGF-like) repeat domains. Each EGF-like domain is rich of cysteine and glycine to produce three loops that are essential for high-affinity binding to its ligand. Nearly all reported CADASIL-associated mutations result in gain or loss of a cysteine residue within the EGF-like domains. Only a few cysteine-sparing *NOTCH3* mutations have been documented in the patients with CADASIL to date. Here, we reported a Chinese CADASIL family with a cysteine-sparing *NOTCH3* mutation. In this family, affected patients had dizziness, memory loss, gait instability, or hemiplegia. Brain magnetic resonance imaging (MRI) showed diffuse leukoencephalopathy with confluent signal abnormalities in the periventricular white matter, basal ganglia, and centrum semiovale bilaterally. By screening the entire coding region of *NOTCH3*, a novel missense mutation p.G149V (c.446G>T) was found. This mutation was not detected in 400 normal controls. Considering the critical position of glycine within the C-loop of EGF-like domain and its high conservation through evolution, p.G149V mutation could be a potential pathogenic cause for CADASIL.

## Introduction

Cerebral autosomal dominant arteriopathy with subcortical infarcts and leukoencephalopathy (CADASIL, OMIM 125310) is an inherited small cerebral vessel disease characterized by migraine, recurrent stroke, mood disturbances, apathy, dementia, and premature death [1], [2]. CADASIL is caused by the mutations in the *NOTCH3* gene (OMIM 600276), which encodes a single pass trans-membrane protein with an extracellular epidermal growth factor-like (EGF-like) repeat domain, a single trans-membrane domain, and an intracellular ankyrin domain [3], [4]. Each EGF-like domain contains six cysteine residues to form three loops, which are essential for the high-affinity for ligand binding. To date, more than 200 mutations of *NOTCH3* have been reported in CADASIL patients. Among these, over 95% mutations are located in EGF-like repeat domain of NOTCH3 [data from HGMD]. The vast majority of mutations are missense and lead to either gain or lose a cysteine residue, resulting in an odd number of cysteine and further misfolding of the EGF-like repeat domain. This misfolding may alter the maturation, targeting, degradation and function of the NOTCH3 receptor, which is responsible for most phenotypes of CADASIL affected families. Interestingly, only several cysteine-sparing *NOTCH3* mutations have been documented in the patients with CADASIL to date [5], [6].

In this study, we reported a novel cysteine-sparing *NOTCH3* mutation at the nucleotide position 446 (c.446G>T; p.G149V) in a Chinese family with CADASIL.

## Materials and Methods

The study was approved by the Institutional Review Board of Soochow University. Written informed consent forms from the family and 400 healthy blood donors were obtained. The clinical data on family members were obtained through interviews, physical examination, brain magnetic resonance imaging (MRI), and medical reports.

Genomic DNA from peripheral blood mononuclear cells of the proband and her relatives were isolated by the method of phenol-chloroform. All 33 exons and their flanking intronic sequences of gene *NOTCH3* (NM_000435.2) were amplified by polymerase chain reaction (PCR) using DreamTaq Green PCR Master Mix (2X) (Fermentas) or TaKaRa LA Taq with GC buffer (Table 1). After the PCR products were directly sequenced by the Sanger method, DNA sequences were analyzed by comparing to the February 2009 human reference sequence (GRCh37/hg19) with the BLAT tool from the UCSC Genome Browser. The new mutation was checked with online database like HGMD (http://www.hgmd.org/) and the 1000 Genome project (http://www.ncbi.nlm.nih.gov/variation/tools/1000genomes/).

**Table 1:** Information of primers used in PCR amplification for *NOTCH3* gene.

Primer Name	Sequence
E1F	GAATCTCTGGGTCCTCCAGGACTGG
E1R	AGGTGTCCGGCTCTGGGTGTGTAC
E2F	GAGGTTGCCCAAGCCACACACATG
E2R	CACCCTTACTTCCTGTCTCAGCACAC
E3-6F	CACTCAAGTCAGACTTCTTATTTGCCCTC
E3-6R	TGCGTGTTTCTTGCCTGTCTTGTG
E7-8F	GACCAAGCCCCCAGAATCACACAG
E7-8R	GGCAGAGCAGGAAGATCTGCCTATGAC
E9-10F	CTGTCACTGGGTCCTGCCTTGCTAC
E9-10R	AGTGTAGAGGTGGGGACGAAGGCTC
E11-2F	GCCAATGAGACAGCACAGACTCAGG
E11-2R	ATTGGTCCGAGGCCTCACTTGTG
E13-5F	GCTGACCCACACAGGCTGCATATC
E13-5R	GACTTCATGGAAGTTTGGGGACCAG
E16F	GCTCATTCCCTAACTCGTTCCATGG
E16R	CACTGATATGCAGCCTGTGTGGGTC
E17-8F	GTCTGGAGGGGAAGCACTCAGAGTC
E17-8R	TCCTGCCCTTGCCACATAGGTGAG
E19-20F	CCAGATCTACCCAGAGTGACACCCAC
E19-20R	AGTGCCAGGTGGGTGGAGTTACTGG
E21-3F	CAGAAACTCCCTTCCCTTCGATGTC
E21-3R	GTGCTGGGGTTCTTTGCGTCTTC
E24F	GACACGTGGGACACACCGATG
E24R	CCCCTCTCTCCCCTTGACTCTTC
E25F	CAAGCCACTTAGCAACCTGCCTCC
E25R	GGCATGGGTCCGTATATTCCAGTCC
E26-8F	CTGATCACGCCCATCATCCAC
E26-8R	AGATGGCGCTTCAGAGCCAGG
E29F	GGACCTTTGTCACTTCCAACCAAGAG
E29R	TTCCCAGGGCTCTGTGTGTATCTCC
E30-1F	TGAGGCACCCTAAGGACTAGTGGTG
E30-1R	CCCTGTGCCCTAGGAGTAGTTCTGTG
E32F	ACTGCTGACACCCAGTGGACCAAG
E32R	GCACACACATGGATCCAGACACAAG
E33F1	AAGGCAAGGATGCAGGAGGGTTG
E33R1	CTGACAGCTCGGTCACGCTGTC
E33F2	CAAGCTGTGCCAGAGACACTGCAG
E33R2	CCCAAGAGGCTGGAAGACTTTGCTAC
MscI mutF	CAGCTGCGGCCCTGGTgG
MscI mutR	ACTCACCCTGTCCTGGTCCCTCC

All family members and unrelated 400 control individuals were screened by restriction enzyme FastDigest MscI (Fermentas, USA), which recognizes TGG/CCA sites generated with mutagenesis primers (Table 1) in wild type allele but not c.446G>T mutant allele of *NOTCH3* gene.

## Results

### Patient history and clinical features

**Figure 1:**
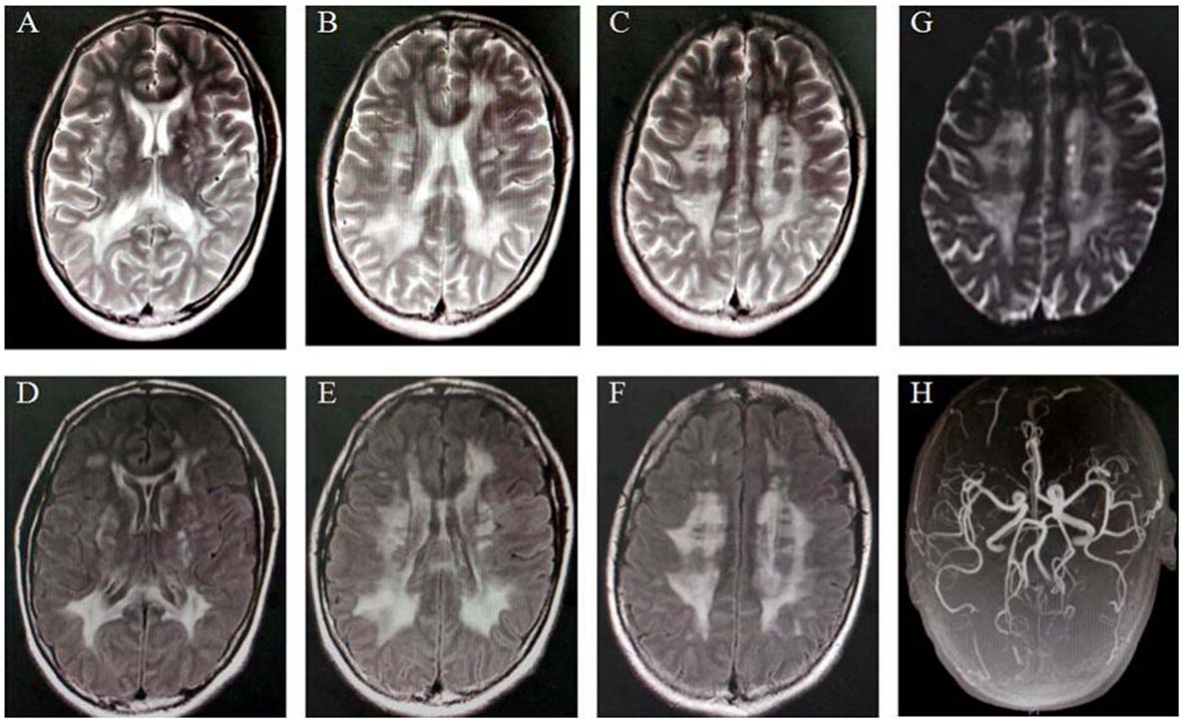
Brain MRI images of the proband showing characteristic diffuse leukoencephalopathy. T2-weighted MRI images (A–C) showed diffuse hyperintensities in the periventricular white matter with a symmetrical distribution, the centrum semiovale, and the basal ganglia. Fluid-attenuated inversion recovery (FLAIR) T2 sequences (D–F) revealed confluent white-matter lesions in multiple brain regions. Diffusion-weighted imaging (DWI) sequence (G) demonstrated acute ischemic stroke in the centrum semiovale. MR angiography (H) revealed no intracranial arterial stenosis or occlusion.

The proband of the family was a 43-year-old woman. At the 39 years of age, she complained progressive dizziness and memory impairment. She had no remarkable medical history. Cerebrovascular risk factors including hypertension, high glucose, hypercholesterolemia, and smoking were not found. Her neurological examination was unremarkable. Her performance on the mini-mental status examination (MMSE) was slightly impaired (a score of 25 out of 30). T2-weighted and fluid-attenuating inversion recovery (FLAIR) brain MRI (GE Healthcare Signa EXCITE 3.0T) revealed severe diffuse leukoencephalopathy with multiple confluent signal abnormalities in the periventricular white matter, basal ganglia, and centrum semiovale bilaterally (Fig. 1A–C and D–F). Diffusion-weighted imaging (DWI) sequence demonstrated acute ischemic stroke in the centrum semiovale (Fig. 1G). Cerebral magnetic resonance angiography (MRA) did not reveal segmental stenosis of the intracranial vessels, vascular malformation, or aneurysm (Fig. 1H). The patient was diagnosed as Binswanger’s disease. At the two-year follow-up, she still had dizziness and hypomnesia. MMSE score was 24 and MRI did not show any significant change in comparison with previous record. The patient reported that her elder sister had similar symptoms and MRI changes. Therefore, her family members were interviewed and clinical data were collected. On the basis of the family history and neuroimaging data, a diagnosis of CADASIL was suspected.

**Figure 2:**
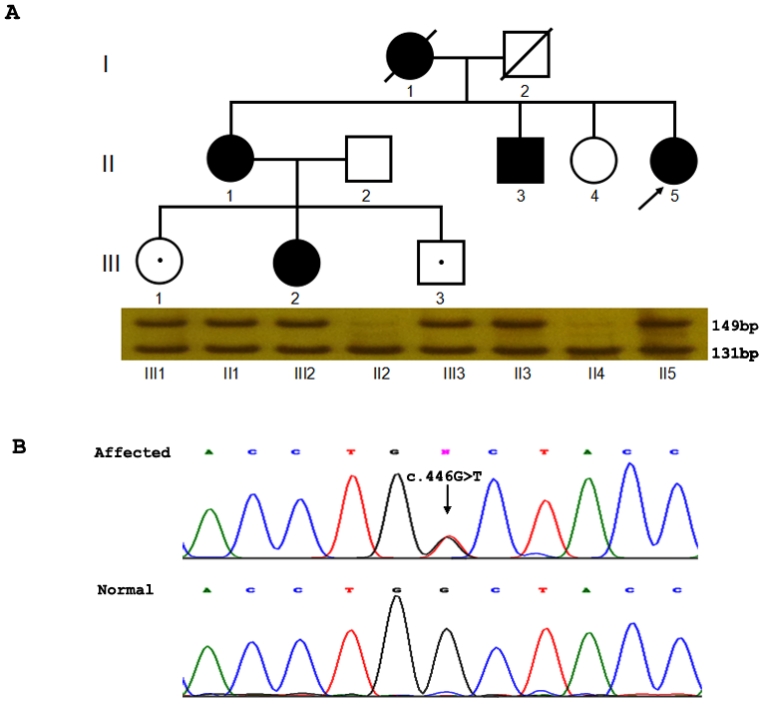
A novel G149V mutation found in a CADASIL family. The pedigree of this Chinese CADASIL family was shown (A). The proband is indicated with an arrow. MscI digestion results corresponding to each individual were shown below the pedigree. The affected showed a digested band (131 bp) with an undigested band (149 bp), while the normal controls showed only one digested band at 131 bp. III1 and III3 carried the mutation but are asymptomatic so far. Electropherograms of the sequence showed the nucleotide change in the affected individual with respect to the normal control bp. III1 and III3 carried the mutation but are asymptomatic so far. Electropherograms of the sequence showed the nucleotide change in the affected individual with respect to the normal control (B). P.G149V (c.446G>T) was indicated with an arrow.

The family pedigree was draw as in Fig. 2A. The elder sister of the proband (II1) initially presented dizziness, hypomnesia, and gait disability at 48 years of age. She was admitted for further examination at that time. She had no history of hypertension and diabetes mellitus. Her neurological examination was unremarkable except for bilateral positive Babinski sign, positive Romberg test, and mild memory disturbance (the MMSE score 23). Brain MRI indicated that multiple confluent hyperintensities were observed in the periventricular white matter, basal ganglia, and centrum semiovale. Her symptoms including memory loss were progressive. She gradually had the difficulty to chew, talk, and swallow. At the age of 52 years old, the patient was feed by a gastrostomy tube and was extremely thin so that she could not move. The MMSE score was 2. Neurological assessment revealed high muscle tone and brisk tendon reflexes in limbs.

The elder brother of the proband (II3) was diagnosed as ischemic stroke for hemiplegia at 40 years of age. He did not have any vascular risk factors and was partially recovered within several months. The niece of the proband (III2) had dizziness at 26 years of age, but no neurological abnormality was found.

### Genetic analysis

We first screened the exons 2–24, which encode 34 EGF-like repeat domains of *NOTCH3* gene of the proband by direct sequencing. We found a novel G to T transversion at the nucleotide position 446 (c.446G>T), resulting in a change of glycine (GGC) to valine (GTC) at the amino acid position 149 in *NOTCH3* (Fig. 2B). We further screened the remaining exons of *NOTCH3* gene and no other potentially pathogenic nucleotide change was detected. The c.446G>T change causes a loss of MscI restriction enzyme site (TGG/CCA) in the mutant allele from the 149 bp PCR product amplified by the mutantgenesis primers which were designed to contain C at nucleotide position 448 instead of T. By MscI enzyme, the heterozygous c.446G>T mutation was found in all the affected individuals and two young asymptomatic individuals (III1 and III3) in the family (Fig. 2A). Additional 400 health controls have been screened by MscI digestion, however, this c.446G>T mutation was not found in these individuals. There was no report about this c.446G>T change in HGMD and the 1000 Genome project database.

**Figure 3:**
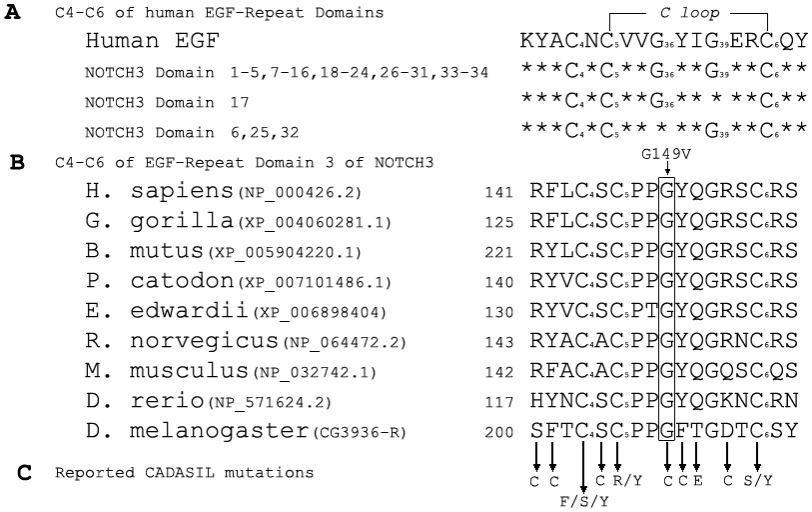
Alignment of EGF-like repeat domain of NOTCH3. Alignment of C4–C6 in EGF-like domains of NOTCH3 was shown in the upper panel (A). Conserved G36 in human EGF is shown in the most EGF-like domains of NOTCH3. Alignment of C4–C6 in EGF-like repeat domain 3 homolog from different species was shown in the middle panel of the figure (B). Amino acid sequence alignment from various species showed the conservation of this glycine residue. The reported CADASIL mutations within this EGF-like repeat domain were indicated in the lower panel (C).

The glycine at position 149 is located at the third EGF-like domain and lies between C5–C6 to form the C-loop of NOTCH3. It is corresponding to human EGF Gly36 and is highly conserved in nearly all the EGF-like repeat domains of NOTCH3 except domain 6, 25 and 32 (Fig. 3A). The glycine 149 lies in a center of a clusters reported mutations and shows high conservation through evolution from human to fly (Fig. 3B, 3C).

## Discussion

In this study, we reported a novel *NOTCH3* gene missense mutation (p.G149V) in a Chinese CADASIL family. The clinical features in this family included dizziness, cognitive deficits, gait instability, and ischemic stroke. All affected patients had no vascular risk factors. MRIs revealed T2-weighted hyperintensities in the periventricular and subcortical white matter. Based on family history, clinical symptoms, and typical MRI results, CADASIL was diagnosed.

CADASIL-causing protein NOTCH3 has 34 EGF-like repeat domains [7], [8]. Six highly conserved cysteine residues within each EGF-like domain form three intra-molecular disulfide bond pairs between C1–C3, C2–C4 and C5–C6. These three loops are essential for high-affinity binding to the ligand. When ligands bind to the extracellular EGF-like repeat domain, extracellular domain was released with a series of proteolytic cleavages, then translocates to the nucleus, and activates transcription. Mutations within EGF-like repeat domain alter the conformation of the receptor and lead to defects in NOTCH3 protein processing and trafficking, or interaction with the ligands [9]. As the second rich amino acid next to cysteine, glycine showed high degree of conservation at Gly18, Gly36, and Gly39 of human EGF (hEGF) domain. By genetic testing, we revealed a novel c.446G>T (p.G149V) mutation located in exon 4 of the *NOTCH3* gene. The glycine at position 149 is located at the third EGF-like domain of NOTCH3 between C5–C6. This p.G149 is corresponding to the Gly36 of hEGF and is also highly conserved among almost all the EGF like domains of NOTCH3 except domain 6, 25 and 32 (Fig. 3A). Mutagenesis studies showed that the substitute of Gly36 by valine resulted in an apparent inability of EGF to fold into its native structure [10], which make it more easily to predict that G149V mutation might dysfunction the critical role of glycine and cause the phenotype of CADASIL. Similarly, the mutation c.445G>T (p.G149C) was reported in 5 individuals with CADASIL [11], which supported our hypothesis that the institution of glycine at position 149 of *NOTCH3* is a potential pathogenic cause of CADASIL. In addition, this mutation was not found in 400 health controls and no other mutation of *NOTCH3* gene is responsible for the phenotype of CADASIL, as further supported this notion.

To date, only a few cysteine-sparing missense mutations within EFG-like repeat domains have previously been reported as the cause of CADASIL. Four of them (R75P, Q151E, T577A, P685T) are located within C-loop of EGF-like domain (data from HMGD). It might be due to the hypothesis that C-loop is more likely to be responsible for the binding affinity since it is more conservative than the other two loops [12].

In summary, we found a novel cysteine-sparing *NOTCH3* mutation G149V in a family with CADASIL. Considering its critical position of glycine within the C-loop of EGF-like domain, p.G149V mutation could be a potential pathogenic cause for CADASIL, which will be further confirmed by functional studies.
